# Comparative Genomics of Pathogenic and Nonpathogenic Strains of *Xanthomonas arboricola* Unveil Molecular and Evolutionary Events Linked to Pathoadaptation

**DOI:** 10.3389/fpls.2015.01126

**Published:** 2015-12-22

**Authors:** Sophie Cesbron, Martial Briand, Salwa Essakhi, Sophie Gironde, Tristan Boureau, Charles Manceau, Marion Fischer-Le Saux, Marie-Agnès Jacques

**Affiliations:** ^1^INRA, UMR 1345 Institut de Recherche en Horticulture et SemencesBeaucouzé, France; ^2^Université d'Angers, UMR 1345 Institut de Recherche en Horticulture et SemencesAngers, France

**Keywords:** *Juglans regia*, vertical oozing canker, bacterial blight, ICE, copper resistance

## Abstract

The bacterial species *Xanthomonas arboricola* contains plant pathogenic and nonpathogenic strains. It includes the pathogen *X. arboricola* pv. *juglandis*, causing the bacterial blight of *Juglans regia*. The emergence of a new bacterial disease of *J. regia* in France called vertical oozing canker (VOC) was previously described and the causal agent was identified as a distinct genetic lineage within the pathovar *juglandis*. Symptoms on walnut leaves and fruits are similar to those of a bacterial blight but VOC includes also cankers on trunk and branches. In this work, we used comparative genomics and physiological tests to detect differences between four *X. arboricola* strains isolated from walnut tree: strain CFBP 2528 causing walnut blight (WB), strain CFBP 7179 causing VOC and two nonpathogenic strains, CFBP 7634 and CFBP 7651, isolated from healthy walnut buds. Whole genome sequence comparisons revealed that pathogenic strains possess a larger and wider range of mobile genetic elements than nonpathogenic strains. One pathogenic strain, CFBP 7179, possessed a specific integrative and conjugative element (ICE) of 95 kb encoding genes involved in copper resistance, transport and regulation. The type three effector repertoire was larger in pathogenic strains than in nonpathogenic strains. Moreover, CFBP 7634 strain lacked the type three secretion system encoding genes. The flagellar system appeared incomplete and nonfunctional in the pathogenic strain CFBP 2528. Differential sets of chemoreceptor and different repertoires of genes coding adhesins were identified between pathogenic and nonpathogenic strains. Besides these differences, some strain-specific differences were also observed. Altogether, this study provides valuable insights to highlight the mechanisms involved in ecology, environment perception, plant adhesion and interaction, leading to the emergence of new strains in a dynamic environment.

## Introduction

Xanthomonads are bacteria associated to plants and are commonly pathogens of plants (Vauterin et al., 2000). These bacteria can infect a wide host range and cause diseases on more than 124 monocot species and 268 dicot species including cereals, solanaceous and brassicaceous plants, stone and nut fruit trees (Hayward, 1993; Vauterin et al., 2000). Symptoms and plant parts affected are diverse, however each strain is characterized by a narrow host range. This has led to the definition of the pathovar concept. A pathovar is a group of strains responsible for the same disease on the same host range (Dye et al., 1980).

*X. arboricola* comprises pathogenic strains distributed in different pathovars (Fischer-Le Saux et al., 2015). The most economically important pathovars in *X. arboricola* are pathovars *pruni, corylina*, and *juglandis*, which affect stone and nut fruit trees. *X. arboricola* pv *juglandis* is the causal agent of walnut blight (WB), a serious disease of Persian (English) walnut. It causes necrosis on leaves, catkins, twigs, and fruits, and can induce important crop losses. A few years ago, a new genetic lineage was identified within *X. arboricola* pv *juglandis* as the causal agent of a new disease called vertical oozing canker (VOC) (Hajri et al., 2010). Nonpathogenic *X. arboricola* strains were also isolated from walnut tree during surveys of French orchards. These strains are unable to cause any disease on walnut tree and other plant species (Essakhi et al., 2015). Such xanthomonads, nonpathogenic strains on their host of isolation, have already been isolated from a range of different plants (Vauterin et al., 1996; Vandroemme et al., 2013a; Triplett et al., 2015). Within *X. arboricola*, nonpathogenic strains from *Juglans regia* and from *Fragaria* × *ananassa* are phylogenetically diverse and do not cluster according to their host of isolation contrary to pathogenic strains from pathovars *pruni, corylina*, and *juglandis* (Vandroemme et al., 2013a; Essakhi et al., 2015; Fischer-Le Saux et al., 2015).

Type three effectors (T3Es) secreted in host plant cells via the type three secretion system (T3SS) play a basic role in pathogenicity and host specificity of xanthomonads (Hajri et al., 2009). It was previously shown that strains causing WB and VOC diseases differ by their T3E repertoires, which is composed of 17 T3Es (Hajri et al., 2012). The strains causing VOC could be differentiated from other strains within the pathovar *juglandis* by the absence of *xopAH* and the presence of *xopB* and *xopAI*, the latter being specific to VOC strains within *X. arboricola*. In contrast, some genetic lineages of nonpathogenic strains are devoided of *hrp*/*hrc* genes encoding the T3SS and possess three T3Es at the most (Vandroemme et al., 2013a; Essakhi et al., 2015). Other nonpathogenic strains possess T3SS genes and seven T3Es genes (*xopR, xvrBs2, avrXccA1, xopA, xopF1, hrpW, hpaA*) among the 18 analyzed T3Es. These results indicate that *X. arboricola* is a model of choice to study the evolutionary events that lead to the emergence of epidemic populations and to decipher the molecular determinants of virulence. Comparative genomic analysis among *Xanthomonas* are useful to identify the distinct gene contents related to virulence, to reveal new features and to explain the differing pathogenic processes (Ryan et al., 2011).

In this report, we present genomic comparisons of four *X. arboricola* strains isolated from walnut tree that are representative of the bacterial diversity encountered on *J. regia* and that were previously analyzed by MLSA, MLVA, and T3Es repertoire (Hajri et al., 2012; Essakhi et al., 2015; Fischer-Le Saux et al., 2015). Strain CFBP 2528 (the type strain of the species), which causes WB, strain CFBP 7179, which causes VOC, both included in the pathovar *juglandis* and two nonpathogenic strains, CFBP 7651 and CFBP 7634, isolated from healthy walnut buds and representing two genetic lineages of nonpathogenic strains with and without *hrp/hrc* genes coding the T3SS respectively were chosen (Essakhi et al., 2015). The aim of this work is to identify differences between pathogenic and nonpathogenic strains, in order to unveil mechanisms of emergence of pathogenic strains. Based on genomic results, phenotypic tests were conducted in an attempt to link genomic content to phenotypic features.

## Materials and methods

### Bacterial strains

Bacterial strains used in this study are listed in Table S1. Strains of *X. arboricola* were obtained from the International Center for Microbial Resources, French Collection for Plant-associated Bacteria, (CIRM-CFBP), INRA, Angers, France (http://www.angers.inra.fr/cfbp/) or isolated from buds of healthy walnuts in the two main walnut-growing areas in France (Rhône-Alpes region in the southeast and Périgord in the southwest). Bacterial strains were routinely grown at 27°C on TSA medium (3 g/l trypton soya broth; 10 g/l agar) for 24–48 h.

### Genomic DNA isolation, sequencing, and annotation

Genomic DNAs from the strains CFBP 2528, CFBP 7179, CFBP 7634, and CFBP 7651 were isolated and purified using the Qiagen's genome DNA isolation kit (Qiagen, Hilden, Germany) according to the manufacturer's instructions. The Genomic DNA quality and quantity were assessed on an agarose gel and using a NanoDrop ND-1000 spectrophotometer (the NanoDrop Technologies, Wilmington, DE). Libraries with an average insert size of 250 bp and 3 kb (mate- pair libraries) respectively were sequenced using the Illumina HiSeq 2000 platform (GATC Biotech, Germany). Paired-end reads were assembled in contigs using SOAP*denovo* 1.05 (Li et al., 2010) and Velvet 1.2.02 (Zerbino and Birney, 2008). Then contigs were scaffolded with LYNX (Gouzy, unpublished data) using mate-pair read information. Annotation was performed using EuGene-PP using similarities with known protein sequences (Sallet et al., 2014). A probably non-exhaustive list of known T3Es that were previously identified in various pathogenic bacteria genus (*Xanthomonas, Pseudomonas, Ralstonia, Erwinia, Escherichia, Salmonella*) was used to screen for homologs of these effectors in the four *X. arboricola* genomes using tBLASTN and BLASTP. Sequences displaying high sequence similarity (observed with % of length and % identity) with any previously described T3E were searched. We also searched the presence or absence of T3E genes screened by Hajri et al. (2012). The two models “T4SEpre_bpbAac” and “T4SEpre_psAac” of the T4SEpre package (Wang et al., 2014) were used to predict type four effectors (T4E) from the four *X. arboricola* genomes. Type six secretion system (T6SS) genes from *Xanthomonas campestris* pv *vesicatoria* 85-10, *Xanthomonas fuscans* subsp. *fuscans* 4834R, *Xanthomonas oryzae* pv *oryzae* PXO99A, were used for BLASTN against the four *X. arboricola* genomes.

### Genome accession numbers

The *X. arboricola* genome sequences of strains CFBP 2528, CFBP 7179, CFBP 7634, and CFBP 7651 have been deposited at DDBJ/EMBL/GenBank under accession no. JZEF00000000, JZEG00000000, JZEH00000000, and JZEI00000000, respectively.

### Genomic comparisons

Identification of orthologous groups between genomes was achieved by orthoMCL V2.0.9 analyses on predicted full-length protein (Li et al., 2003). OrthoMCL clustering analyses were performed using the following parameters: *P*-value Cut-off = 1 × 10^−5^; Percent Match Cut-off = 80; MCL Inflation = 1.5; Maximum Weight = 316. We modified OrthoMCL analysis by using −F′m S′ option during the BLASTP pre-process. From results were defined unique CDSs, corresponding to CDSs present only in one copy in one genome, and groups of orthologs that corresponded to CDSs present in one copy in at least two genomes. The main part of comparative analyses of genomes and figures were deduced from their distribution. Furthermore, genomes contained CDSs that were present at least in two copies (paralogs) in one or more genomes. Groups of homologs referred to groups of orthologs having paralogs. Venn diagram were obtained using the R package ≪ VennDiagram ≫ 1.6.5. Chromosomal rearrangements were explored using a script adapted from the R package ≪ genoPlotR ≫ 0.8.2 (Guy et al., 2010). A circular representation of the orthoMCL analysis was generated with the CGView tool (Grant and Stothard, 2008).

### Phylogeny

Average Nucleotide Identity (ANI) analysis was performed as in Scortichini et al. (2013). The Composition Vector Tree (CVTree) tool (Xu and Hao, 2009) was used to build a phylogenetic tree with the four genomes sequenced in this study and eight *X. arboricola* genomes available in public databases (four *X. arboricola* pv *pruni* strains: MAFF 301427, MAFF 301420, MAFF 311562, Xap 33; one *X. arboricola* pc *corylina* strain: NCCB 100457; one *X. arboricola* pv *juglandis* strain: NCPPB 1447; two *X. arboricola* pv *celebensis*: NCPPB 1630, NCPPB 1832). The *X. campestris* pv *campestris* ATCC 33913 strain was used as an outgroup.

### Prophages detection

PhiSpy algorithm was used to find prophages sequences on the four genomes (Akhter et al., 2012).

### Copper resistance

Bacterial suspensions were standardized to 1 × 10^8^ CFU/ml then spotted (10 μl) in triplicate on CYE-glycerol medium (casitone 1 g/L; yeast extract 0.35 g/L; glycerol 2 ml/L, agar 12 g/L; pH = 7.2), a low nutrient medium with limited copper ion binding capacity (Zevenhuizen et al., 1979), supplemented with Cu^++^: 0 (control), 4, 8, 16, 32, 64 μg/ml. Copper was brought under CuSO_4_, 5H_2_O form. Cultures were incubated at 28°C for 72 h. The minimum inhibitory concentration (MIC) in Cu^++^ that prevented colony growth was recorded. Strains able to grow on 32 μg/ml or greater were considered copper resistant (Gardan et al., 1993).

### Design of PCR primers for analysis of copper resistance genes

Primer pairs were designed with Primer3 (Koressaar and Remm, 2007; Untergasser et al., 2012) on 11 genes including copper resistance genes (Table S2) located on a CFBP 7179 strain-specific cluster. PCR assays were performed in 20 μl volumes containing 62.5 μM dNTP, 0.125 μM each primer (Table S2), 4 μl of GoTaq 5 X buffer, 0.3 U/μl of GoTaq polymerase, and 5 μl of a boiled bacterial suspension (1 × 10^7^ CFU/ml). PCR conditions were 3 min at 94°C; followed by 35 cycles of 30 s at 94°C, 30 s at annealing temperature specific of each primer pair, an elongation time adapted to amplicon size at 72°C; and ended with 10 min at 72°C. PCR amplifications were performed in duplicate for each strain.

### Pectinase assays

Bacterial suspensions were standardized to 3 × 10^8^ CFU/ml then spotted (20 μl) in triplicate on plates. Pectate lyase and Polygalacturonase activities were determined on agar (12 g/L) plates containing polygalacturonic acid (5 g/L) (Sigma) as substrate in Tris-HCl 0.05 M, pH = 8.6 supplemented with CaCl_2_ 0.5 mM or in citrate-NaOH 0.1 M, pH = 5, respectively. After 1 week incubation at 28°C, the plates were flooded with Cetyl TrimethylAmmonium Bromide (CTAB) (Eurobio) 1% overnight. Pectate Lyase and Polygalacturonase activities appeared as translucent halos around colonies. Pectin methyl esterase activity was determined on agar (12 g/L) plates containing pectin from citrus (5 g/L; 85% esterified; Sigma) as substrate in citrate-Na_2_HPO_4_ 0.1 M, pH = 6.4. Plates were flooded with malic acid 0.1 M (Sigma) during 1 h and then stained with ruthenium red 0.02% (Sigma) overnight. Pectin Methyl Esterase activity appeared as a dark red halo surrounding the colonies. All tests were done twice.

### Motility tests

Strain motility was tested in soft-agar assays as detailed in Darrasse et al. (2013). Xanthomonad strains were grown at 28°C up to 12 days in MOKA (yeast extract 4 g/l; casamino acids 8 g/l; KH_2_PO_4_ 2 g/l; MgSO_4_.7H_2_O 0.3 g/l) and TSA 10% (tryptone soja 3 g/l) medium containing 2 g/l agar. A drop (10 μl) of a 1 × 10^8^ cfu/ml suspension was deposited in the middle of the plate and the plates were imaged at 5 days. Two independent experiments with three replicates each were conducted.

### Design of PCR primers for analysis of flagellar cluster

To analyse the flagellar cluster diversity, primers developed by Darrasse et al. (2013) to amplify *fliM, fliE, fliC*, and *flgE* were used in the same conditions. Primer pairs to amplify *fleQ* and *flgB* were designed on CFBP 7179 genome sequence (Table S2).

## Results

### General features of the genome sequences

**Figure 1:**
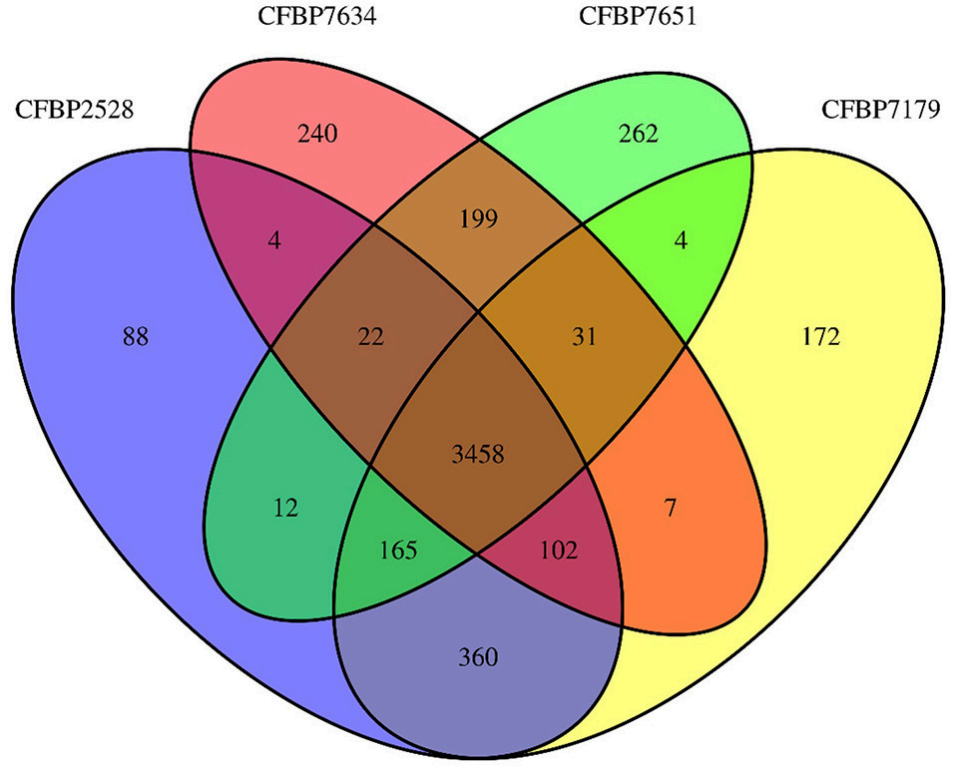
**Venn diagrams illustrating the comparisons of ***Xanthomonas arboricola*** genomes**.

**Figure 2:**
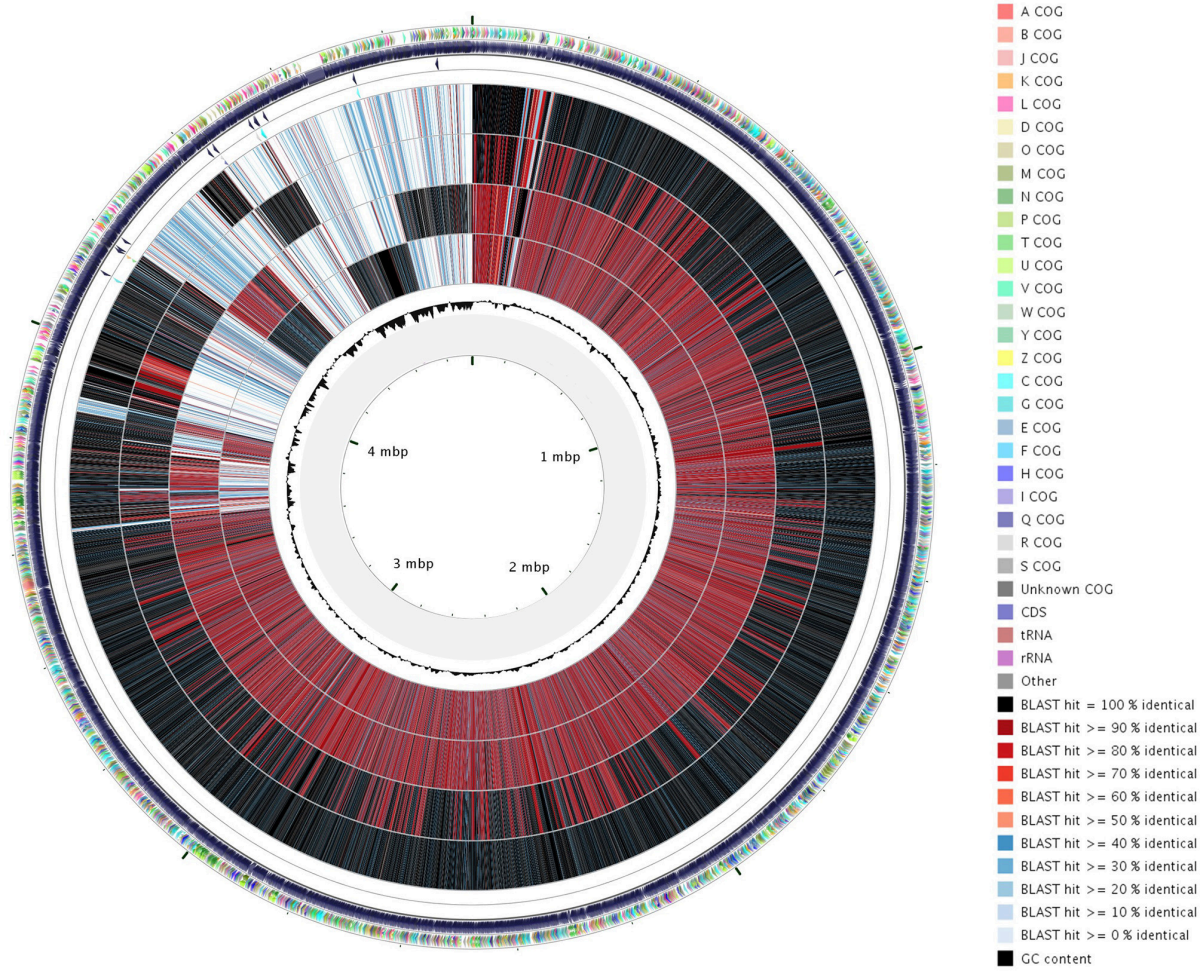
**Circular representation with CGView tool of the orthoMCL analysis of the four ***X***. ***arboricola*** genomes**. Genomic sequences were compared against each other and BLAST results were converted in a graphical map showing the entire sequences. From inside to outside, circle 1 indicates the G+C content, circles 2 to 5 represent CFBP 7634, CFBP 7651, CFBP 7179, CFBP 2528 locus-tags. The external circle shows COG classification of CDSs with different colors according to the legend. In circles 2 to 5, the color indicates the BLAST score (see legend).

General features of the genomes sequenced are summarized in Table 1. The sequencing yielded about 57–71.6 million reads giving approximately 583- to 733-fold theoretical genome coverage. The assemblies had a total length comprised between 4.93 and 5.16 MB with the lowest numbers of scaffolds obtained for the nonpathogenic strains (Table 1). The G+C contents of CDS ranged from 35 to 77% with an average varying between 65.92 (CFBP 2528) and 66.04% (CFBP 7634) (Table 1). Annotation of the genome sequences revealed between 4141 (CFBP 7634) and 4399 (CFBP 7179) putative protein-coding sequences (CDSs), 1 (CFBP 7634) to 12 (CFBP 2528) pseudogenes, 52 or 53 tRNA, and one rRNA operon. We noticed that an extra 16S rRNA gene was present in CFBP 7179. The two 16S rRNA copies exhibited less than 80% identity. The four genome sequences of *X. arboricola* totalized 5126 ortholog groups and specific CDSs of each strain (Figures 1, 2). Of those sequences, 3383 (66% of the CDSs) have been assigned to putative functions based on homology with other known proteins and functional domain analysis. No extrachromosomal plasmid have been detected in any of the four strains.

**Table 1:** **General features of the four draft genome assemblies**.

**General features**	**CFBP 2528**	**CFBP 7179**	**CFBP 7634**	**CFBP 7651**
Size (pb)	5,089,543	5,161,669	4,935,785	5,032,592
G+C, %	65.92	65.93	66.04	65.98
N50 of contig size (pb)	1,295,080	933,664	3,063,886	4,192,770
Number of scaffolds	8	14	4	6
Average coverage	607	733	661	583
CDS, total	4325	4399	4141	4229
tRNA	53	52	52	53
number of ribosomal operon	1	1^a^	1	1
Pseudogenes	12	9	1	3

a *Presence of an extra 16S rRNA gene*.

### Phylogenomic relationships among completely sequenced *Xanthomonas* strains

**Figure 3:**
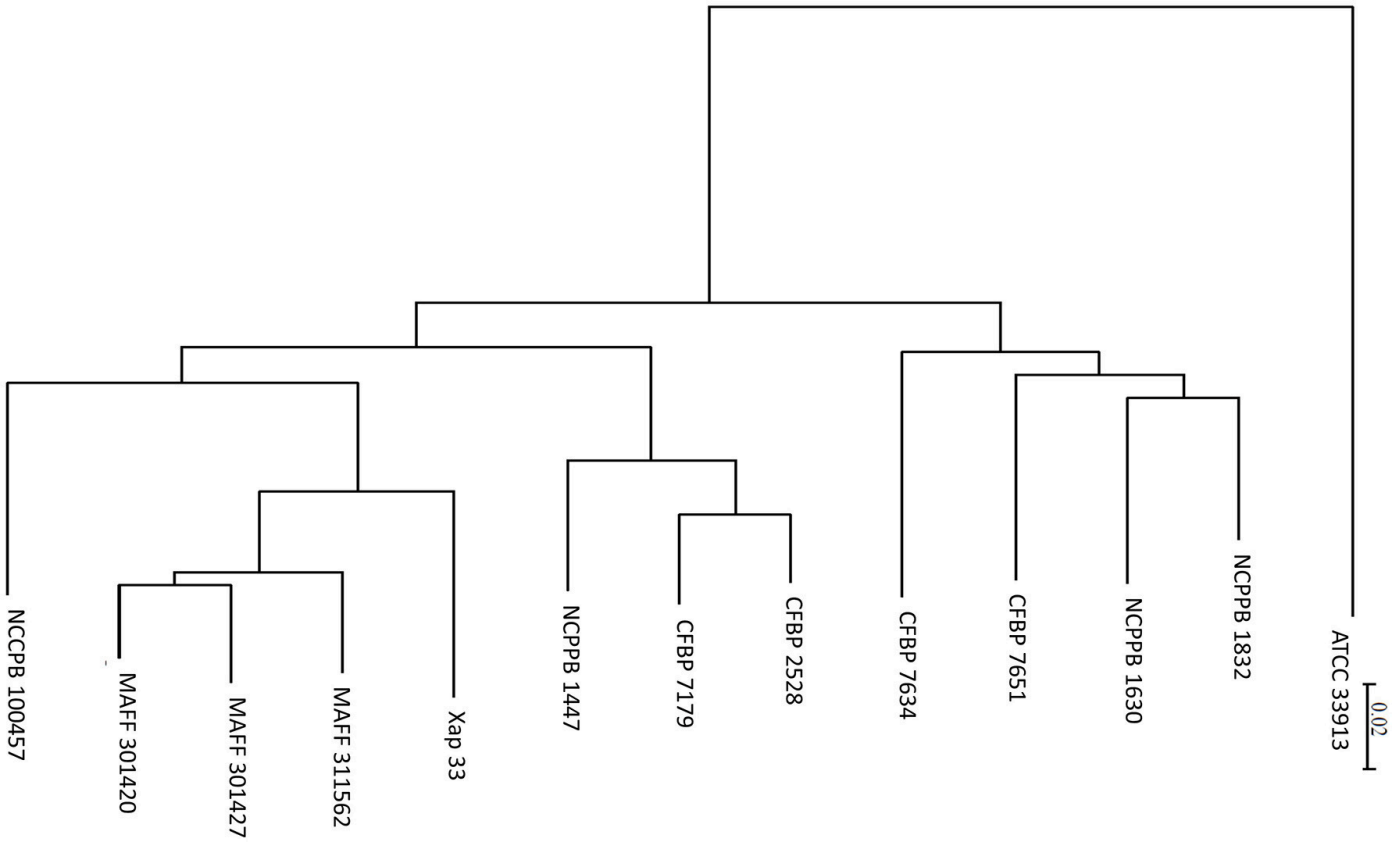
**CVTree obtained from ***X. arboricola*** genome sequences available in public database**. *Xanthomonas campestris* pv *campestris* (ATCC 33913); *Xanthomonas arboricola* pv celebensis (NCPPB 1832, NCPPB 1630); *Xanthomonas arboricola* (CFBP 7651, CFBP 7634); *Xanthomonas arboricola* pv *juglandis* (NCPPB 1447, CFBP 2528, CFBP 7179); *Xanthomonas arboricola* pv *pruni* (Xap 33, MAFF 311562, MAFF 301427, MAFF 301420); *Xanthomonas arboricola* pv *corylina* (NCCPB 100457). Branch length represents the distance calculated using alignment free composition vector method.

We used the CVTree tool to study phylogeny of whole genome sequences from *X. arboricola* strains available in public databases. The tree obtained by this algorithm (Figure 3) showed that stone and nut fruit tree pathogens clustered according to pathovar classification and shared the same phylogenetic origin. The nonpathogenic strains were included in a different clade together with *X. arboricola* pv *celebensis*, a pathovar of minor incidence (Fischer-Le Saux et al., 2015). The ANI values were all above 95, ranging from 96.4 to 96.7, except between strains CFBP 2528 and CFBP 7179, for which the value was higher (99.2).

### A differential repertoire of insertion sequences (IS) elements is observed between pathogenic and nonpathogenic strains

In their most basic form, IS elements consist of a single gene coding for a site-specific recombinase (called a transposase) and short terminal inverted repeat sequences that are recognized by the transposase. CDSs corresponding to transposases were found to be scattered over the different scaffolds. The number of IS elements (Table S3) was strikingly different between pathogenic and nonpathogenic strains, indeed a total of 45 (CFBP 2528), 42 (CFBP 7179), and only four (CFBP 7634 and CFBP 7651) IS elements were found in the chromosomes of these strains.

Most IS elements in our *X. arboricola* strains belonged to the IS3 and IS4 families (Table S3). IS 200-like and IS111A/IS1328/IS1533 were encountered in the four sequenced strains. BLASTP searches led to the identification of IS 200-like in other *Xanthomonas* species such as *X. hortorum, X. citri, X. gardneri*, and *X. campestris*. IS21 as well as Tn3 transposase were only found in CFBP 7179 and in no other *Xanthomonas* species; these IS were related to *Stenotrophomonas maltophilia* and *Pseudomonas aeruginosa* species by BLASTP searches. Mu-transposase was only found in the two pathogenic strains and showed only 92% identity with protein encountered in *X. campestris*.

### An integrative and conjugative element (ICE) specific of CFBP 7179 not yet described in *Xanthomonas* triggers copper resistance

A genomic island (GI) of 94.8 kb (104 CDSs) was identified specifically in the CFBP 7179 genome sequence (Table S3). This island contained CDSs predicted to be involved in integration and conjugation (integrase/recombinase, pilus formation, excisionase) and was flanked by tRNA^gly^ attachment sites, one being adjacent to an integrase gene. These features are characteristic of what has been termed the “backbone” of integrative and conjugative elements (ICEs) (Burrus and Waldor, 2004). This ICE found in *X. arboricola* pv *juglandis* CFBP 7179 will be referred here to as Xaj-ICE. The most striking feature of this GI was the similarity with GI found in bacteria from different genera. Most CDS (102 out of 104) of Xaj-ICE showed high identity (100% identity on 100% of the length) with genes from *P. aeruginosa* strains and *S. maltophilia* strain D457, which belongs to the *Xanthomonadaceae* familly. Among the genes located by this ICE, we found CDSs that are predicted to affect the phenotype of pathogens since they are involved in copper resistance (*copA, copB, copC, copD, copF, copG, copK*), in acriflavin resistance and in detoxification (arsenate reductase, mercuric reductase, mercury scavenger protein and mercuric transport protein). Homologs of *copA* and *copB* were also found elsewhere in the four genome sequences and were highly conserved when compared with those of *X. arboricola* pv *pruni* (96% identity; 100% similarity) and other *Xanthomonas* species. These homologs showed the best identity/similarity score by BLASTN with *cop*A and *cop*B genes described by Lee (1994) in *X. arboricola* pv *juglandis*.

To determine if the *cop* genes found in *copABCDFGK* cluster were strain specific and were correlated with copper resistance, we searched by PCR for 11 genes dispersed all over the Xaj-ICE (including copper resistance genes) in the four sequenced strains and 57 additional *X. arboricola* pv *juglandis* strains initially used by Hajri et al. (2010). We tested these strains for copper resistance on CYE medium supplemented with different concentrations in Cu^++^. For most strains, signals at the expected size were generated indicating that these strains should harbor the entire *copABCDFGK* cluster. Moreover, these strains were shown to be copper resistant (Table 2). However, no signals were obtained for some PCRs in 24 strains, indicating that some genes should be missing. In these strains, no resistance to copper was observed, except for seven strains (CFBP 1022; 12573; 12580; 12582; 12680; 12707; 12714).

**Table 2:** **Copper resistance and PCR results on flagellar genes and on Xaj-ICE**.

	**Strain numbers**	**Flagellar genes**					**Xaj-ICE**	**Copper resistance**
		***flgB***	***flgE***	***fleQ***	***fliE***	***fliM***		**MIC (ppm)**
1	CFBP 878	+	+	−	−	−	−	16
2	CFBP 1022	+	+	+	+	+	−	32
3	CFBP 2528T = ATCC 49083	+	+	−	+	+	−	8
4	CFBP 2564	+	+	+	+	+	−	8
5	CFBP 2568	+	+	+	+	+	−	8
6	12572	+	+	+	+	+	+	32
7	12573	+	+	+	+	+	−	32
8	12574	+	+	+	+	+	+	32
9	12575	+	+	+	+	+	+	32
10	12576	+	+	+	+	+	+	64
11	12577	+	+	+	+	+	−	16
12	12578	+	+	+	+	+	−	16
13	12579	+	+	+	+	+	+	64
14	12580	+	+	+	+	+	−	32
15	12581	+	+	+	+	+	−	8
16	12582	+	+	+	+	+	−	32
17	12583	+	+	+	+	+	+	32
18	12584	+	+	+	+	+	+	32
19	12585	+	+	+	+	+	+	32
20	12586	+	+	+	+	+	+	32
21	12587	+	+	+	+	+	+	64
22	12588	+	+	+	+	+	+	32
23	12589	+	+	+	+	−	+	32
24	12590	+	+	+	+	−	+	32
25	12591	+	+	+	+	+	−	16
26	12592	+	+	+	+	+	+	32
27	12680	+	+	+	+	+	−	32
28	12681	+	+	+	+	+	+	32
29	12707	+	+	+	+	+	−	32
30	12709	+	+	+	+	+	+	32
31	12710	+	+	+	+	+	−	16
32	12711	+	+	+	+	+	+	64
33	12712	+	+	+	+	+	+	64
34	12713	+	+	+	+	+	+	32
35	12714	+	+	+	+	+	−	32
36	12762	+	+	+	+	+	+	32
37	CFBP7179	+	+	+	+	+	+	32
38	12764	+	+	+	+	−	+	64
39	12765	+	+	+	+	+	+	32
40	12766	+	+	+	+	+	+	32
41	12767	+	+	+	+	+	+	64
42	12768	+	+	+	+	+	+	32
43	12769	+	+	+	+	+	+	32
44	12770	+	+	+	+	+	+	32
45	12771	+	+	+	+	+	+	32
46	12772	+	−	+	+	+	+	32
47	12773	+	+	+	+	+	+	32
48	12774	+	+	+	+	+	+	32
49	12775	+	+	+	+	+	+	64
50	12776	+	+	+	+	+	+	32
51	12777	+	+	+	+	+	−	16
52	12778	+	+	+	+	+	+	32
53	12779	+	+	+	+	+	−	16
54	12780	+	+	+	+	+	−	16
55	12781	+	+	+	+	+	+	64
56	12782	+	+	+	+	+	+	64
57	12783	+	+	+	+	+	−	8
58	12784	+	+	+	+	+	−	16
59	12785	+	+	+	+	+	−	16
60	CFBP 7651	+	+	+	+	+	−	16
61	CFBP 7634	+	+	+	+	+	−	16

### Other mobile genetic elements (MGEs) are also differential between pathogenic and nonpathogenic strains

Other MGEs such as prophages or integrases were also examined (Table S3). Four prophages were detected in the pathogenic strain CFBP 2528 instead of one or two in the three other strains. No prophage was shared between pathogenic and nonpathogenic strains. A higher number of integrases was found in pathogenic strains (11–15 integrases per genome) than in the nonpathogenic strains (seven in each genome). The integron described by Gillings et al. (2005) and Barionovi and Scortichini (2008) in pathovars *pruni* and *juglandis* of *X. arboricola*, was localized in the four genomes downstream of the acid dehydratase gene, *ilvD* (Gillings et al., 2005). The integrase gene *intI* should be functional in CFBP 7179 and was degenerated in CFBP 2528. This gene was also degenerated in CFBP 7634 but has retained an integrase domain. *intI* was absent in CFBP 7651 genome. The cassettes of this integron were all different in the four strains and were mostly composed of genes coding hypothetical proteins.

### One hemolysin is specific of pathogenic strains

Hemolysins are toxins secreted via the type I secretion system (T1SS). Homologous CDSs (XARJCFBP 2528_b07940 and XARJCFBP 7179_a04560) coding for a hypothetical protein with a hemolysin BL-binding component (IPR008414 domain) were identified in both pathogenic strains, CFBP 2528 and CFBP 7179. The protein encoded from this CDS showed 98% identity by BLASTP with protein encountered in *X. campestris*. In nonpathogenic strains no CDS was found at the same location. The genes adjacent to the CDS encoding this hypothetical protein were conserved in the four strains. Other CDS encoding proteins linked to hemolysin secretion were only present in pathogenic strains; they possessed HlyB or HlyD domains (XARJCFBP 2528_a06990 and XARJCFBP 7179_b04000; XARJCFBP 2528_d04670 and XARJCFBP 7179_e04740, respectively). These domains are found in ABC transporter (HlyB) and membrane fusion protein (HlyD) from T1SS (Kanonenberg et al., 2013).

### Plant cell wall-degrading enzymes (PCWDEs) are active in nonpathogenic strains

Orthologs of most CWDEs described in Potnis et al. (2011) and Darrasse et al. (2013) were identified in the four *X. arboricola* genomes except that no orthologs of *xyn30A, xynC*, and *cbhA* (encoding 1,4-β cellobiosidase) were found (Table S4).

A differential repertoire of Type 2-secreted degrading enzymes with various activities (peptidases, pectinesterase, pectate lyase, xylosidase…) was identified between pathogenic and nonpathogenic strains (Table S4). On the one hand, homologs of XFF4834R_chr16290 (putative aminopeptidase), XCC0121 (AAM39440, pectinesterase), and of XCC0122 (AAM39441, pectate lyase), were observed in the two nonpathogenic strains. In the two pathogenic strains, only fragments of XFF4834R_chr16290 and XCC0122 were identified and no remnants of XCC0121 were observed. On the other hand, homologs of XFF4834R_chr05470 (coding a putative secreted protease), of XFF4834R_chr25520 (coding a xylosidase), and of XFF4834R_chr23760 (coding a putative pectate lyase), were observed only in pathogenic strains. The putative pectate lyase was observed in pathogenic strains near a peptidase trypsin-like gene. Both were observed in pathogenic strains instead of a pectinesterase gene (GROUPORTHO4194) in nonpathogenic strains. Homologous genes of XFF4834R_chr11410 (encoding for a putative rhamnogalacturonase B) and XFF4834R_chr03290 (encoding a putative glycoside hydrolase) were present in all strains except in CFBP 7179.

**Figure 4:**
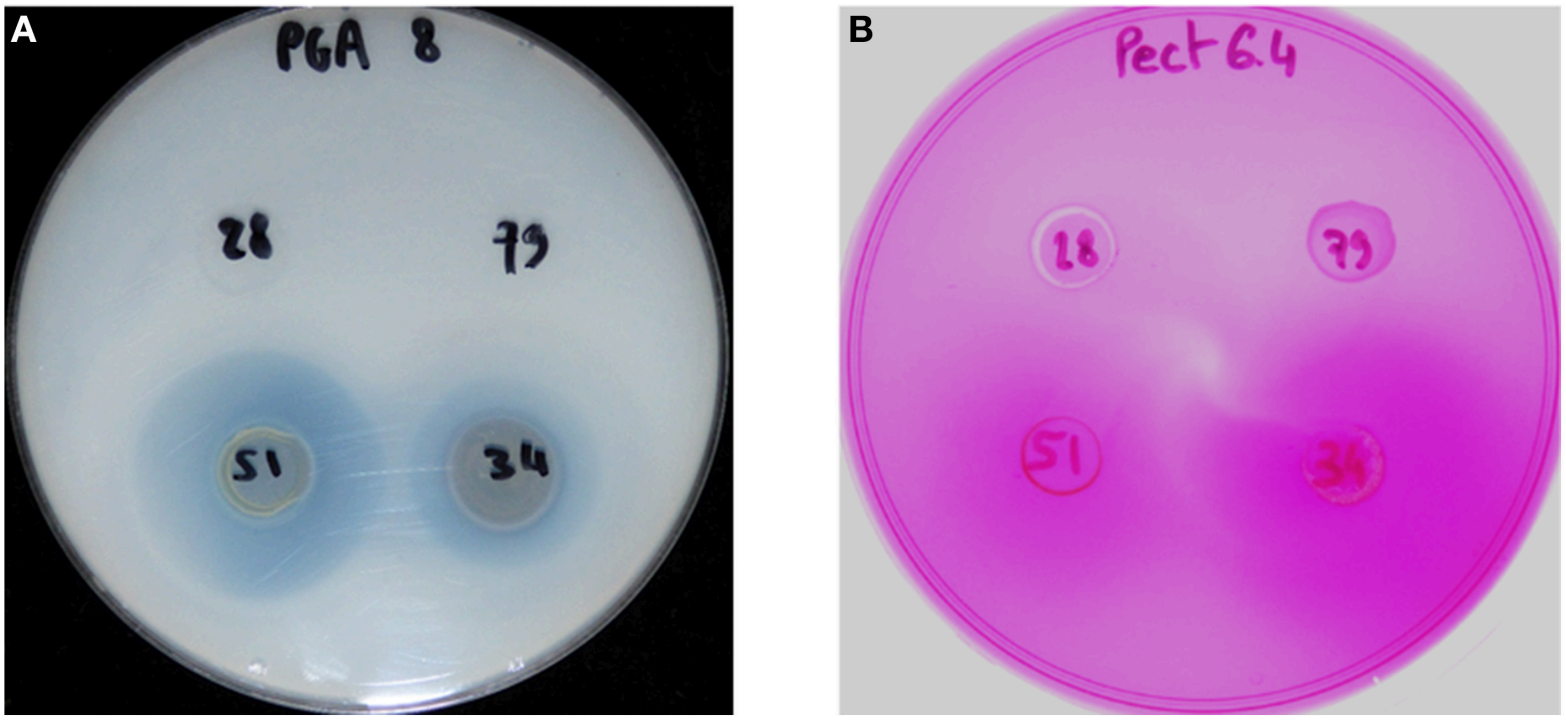
**Detection of pectate lyase activity (A) and pectin methyl esterase activity (B) from the strains CFBP 2528 (28), CFBP 7179 (79), CFBP 7651 (51), CFBP 7634 (34)**. The translucent halo **(A)** and slightly violet **(B)** surrounding the colony indicates the activity.

We compared the pectinase activities of the four strains using plate assays for pectate lyase, polygalacturonase, and pectin methyl esterase. Only the two nonpathogenic strains showed pectate lyase and pectin methyl esterase activities (Figure 4). No polygalacturonase activity was detected for any strain.

### T3SS is absent in the nonpathogenic strain CFBP 7634 and T3Es repertoire is reduced in nonpathogenic strains

**Figure 5:**
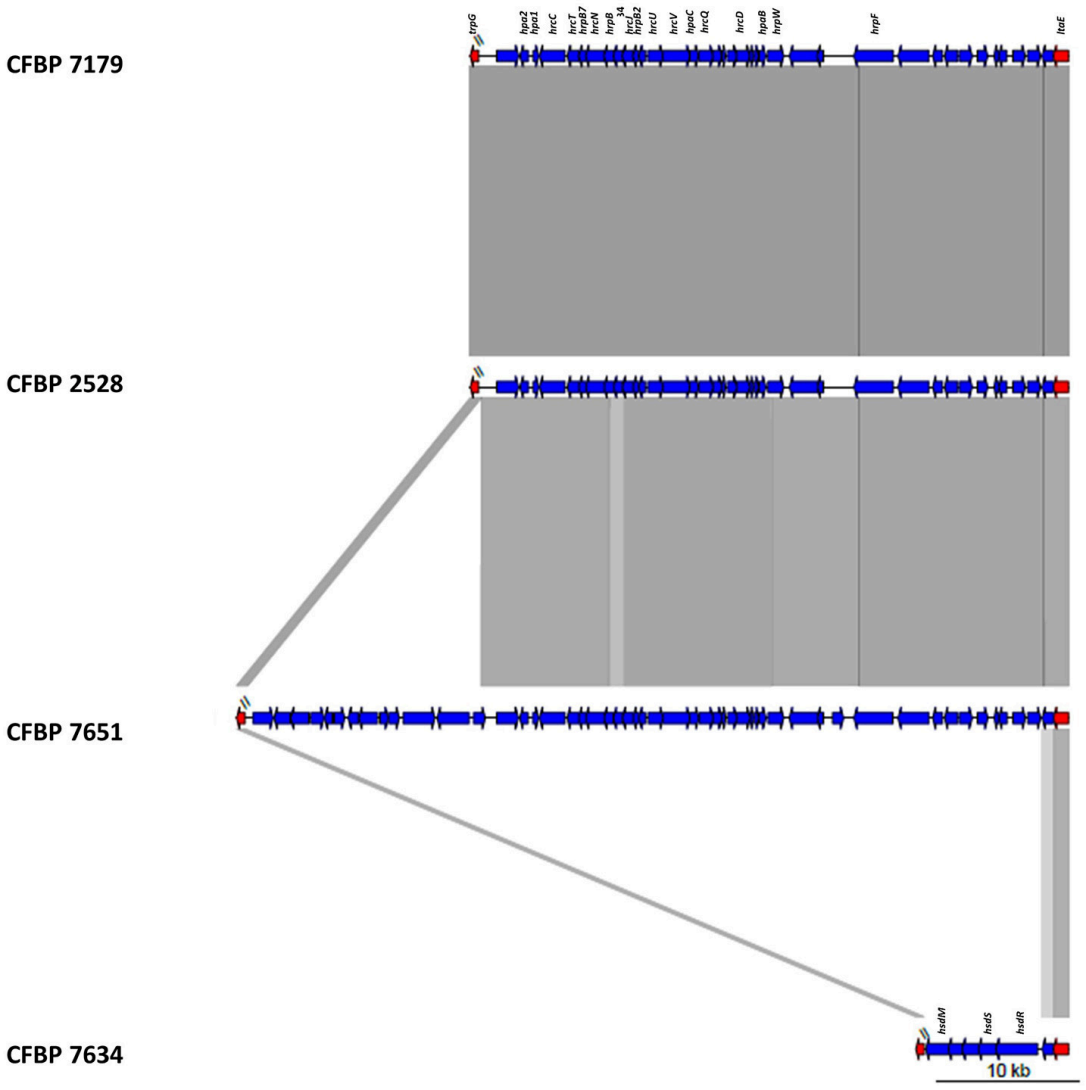
**Schematic representation of the T3SS locus of the four ***X. arboricola*** strains**.

Genomic comparisons of the T3SSs revealed that among conserved genes, the six *hrp* genes (*hrpF, hrpW, hrp D6, hrpB1, hrpB4*, and *hrpB7*), the six *hpa* genes (*hpa1, hpa2, hpa3, hpaA, hpaB, hpaC*) and the 11 *hrc* genes (*hrcC, hrcD, hrcJ, hrcL, hrcN, hrcQ, hrcR, hrcS, hrcT, hrc U, hrcV*) were present in genomes of CFBP 2528, CFBP 7179, and CFBP 7651 and were all absent in the genome of CFBP 7634. The genomic comparisons revealed a high synteny for these clusters in the three strains, with the *hrpF* locus followed by the *hrp/hrc* cluster. The sequences flanking the hrp-island were the *ltaE* gene at the upstream of the *hrpF* peninsula and *trpG* at the downstream of the *hrp/hrc* cluster (Figure 5).

In the nonpathogenic strain CFBP 7634, the *hrp-hrc* region and the *hrpF* peninsula were absent (Figure 5). In fact instead of the *hrp* cluster a region of 8 kb containg genes coding for ATP-dependent restriction enzymes, such as the *hsdR, hsdS*, and *prrC* coding an anticodon nuclease was found between *trpG* and ltaE. These genes compose the type1 Restriction-Modification system known in *Escherichia coli* to be involved in phage defense mechanism (Makarova et al., 2013). A BLAST research with alternative T3SSs observed in other bacteria did not lead to the identification of other T3SS (Araki et al., 2006; Diallo et al., 2012). In CFBP 7651, the *hrp*/*hrc* cluster was followed by a specific region of about 16.7 kb. The first 9 kb are highly identical to regions found in *X*. *c*. pv. *raphani, X*. *c*. pv. *campestris*. This region contained genes encoding a putative xylanase-like, a glycosidase, a methyl-accepting chemotaxis protein (MCP), an oxydoreductase, a transcriptional regulator and a monooxygenase. The last 7.7 kb had BLASTN hits with *Methylobacterium extorquens* and BLASTX hits with dehydrogenase and epimerase implicated in cell envelope biogenesis, two transcription regulators and a sodium/dicarboxylate symporter.

### Examination of the surrounding regions of T3Es provides clues relative to their mechanism of acquisition

Orthologous sets of 24 and 25 genes were predicted in the two pathogenic strains CFBP2528 and CFBP7179, respectively (Table S5). Among these two sets, 17 T3Es genes were already identified by PCR by Hajri et al. (2012) in *X. arboricola* pv *juglandis* and two (*xopAL1* and *xopG*) were not previously identified, probably because of a high diversity in their sequence preventing their amplification by PCR. Two other T3E genes might be present (*xopAA, xopAB*), although the percentage of length was low (74 and 66% respectively). The other genes (*awr4, sfrJ*, and *xopAR*) were not searched by Hajri et al. (2012) and Essakhi et al. (2015). These three genes were predicted in pathogenic and nonpathogenic strains.

Based on the genome sequences, *xopAI* and *xopB* were identified only in CFBP 7179 and not in CFBP 2528. *xopAI*, was close to an IS4 like in CFBP 7179. *xopB* was close to an integrase and an IS4/5. The presence of an integrase close to *xopB* or an IS4 like close to *xopAI* suggested that these T3Es were probably acquired by lateral gene transfer (LGT) in CFBP 7179. In contrast, *xopAH* was identified in CFBP 2528 and not in CFBP 7179 genome sequences as reported by Essakhi et al. (2015) by PCR. Surrounding regions of *xopAH* were the same between strains. This suggests that *xopAH* was probably acquired by homologous recombination in CFBP 2528.

Other T3E coding genes such as *xopN, xopX, xopZ, xopQ, xopK, xopV, xopL, avrXccA2* were scattered in the different scaffolds and were either integrated between genes (*xopN, xopX, xopZ*) that were shared between the four strains, either found in place of a gene shared by the nonpathogenic strains (*xopL, xopV*), or associated to other genes that were not present in the nonpathogenic strains (with transposases for *xopK* or without for *xopQ*).

### The flagellar system is not functional in the type strain CFBP 2528 and the 22-amino-acid flagellin epitope is different in the pathogenic strains

**Figure 6:**
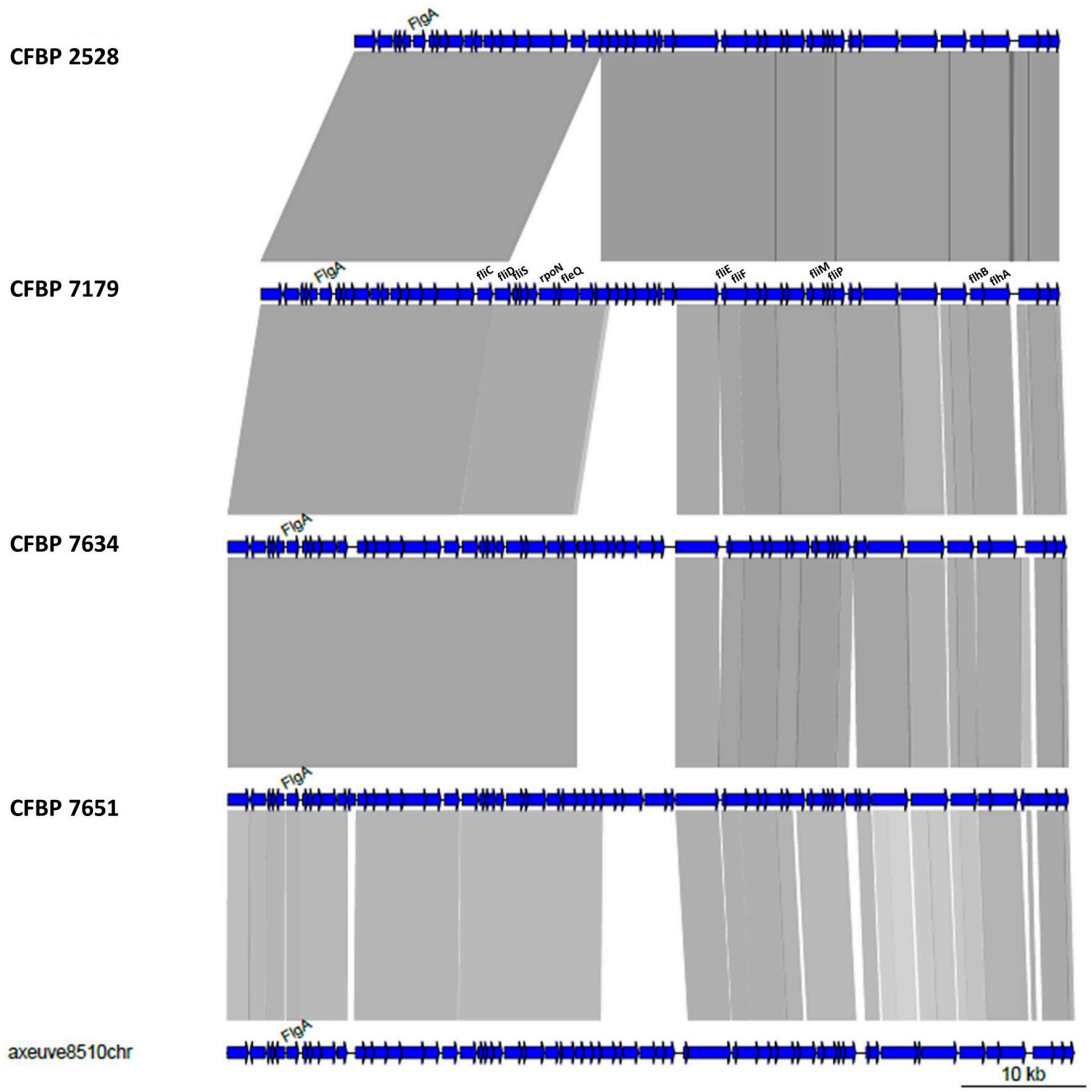
**Schematic representation of the flagellar gene cluster of the four ***X. arboricola*** strains**.

**Figure 7:**
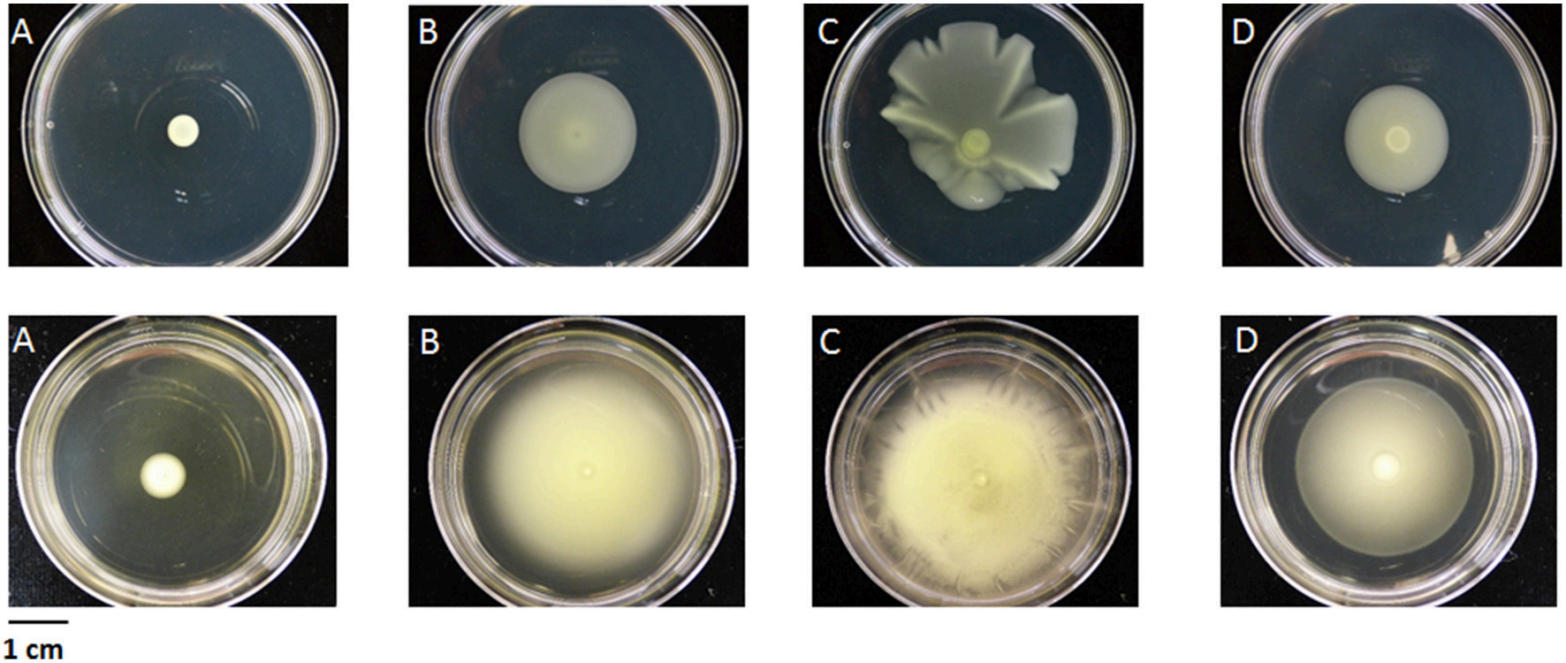
**Motility of the four ***X. arboricola*** strains**. **(A)** CFBP 2528; **(B)** CFBP 7179; **(C)** CFBP 7651; **(D)** CFBP 7634, 60 h after inoculation in TSA 10% (upper line) or MOKA (lower line), with agar 0.2%.

**Figure 8:**
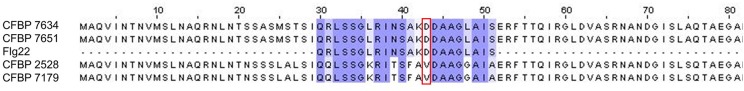
**Multiple alignment between the flagellin FliC N-terminal regions of the 4 ***X***. ***arboricola*** strains and the flg22 peptide**. The “D” to “V” switch between nonpathogenic and pathogenic strains is shown with the red box.

Annotation of the flagellar system reveals that a group of nine contiguous genes was lacking in CFBP 2528 compared to CFBP 7179 genome and genomes of nonpathogenic strains. This group of missing genes included *fliS*, a secretion chaperone for the flagellar filament protein FliC, and *rpoN*, the sigma factor 54 (σ^54^) regulating the flagellar system (Figure 6). No swimming motility was observed for CFBP 2528 in a soft agar-assay (Figure 7). To determine if this event could be observed in other *X. arboricola* pv *juglandis* strains consensus primer pairs were used for PCR-amplification of genes dispersed over the flagellar cluster. A collection of *X. arboricola* pv *juglandis* strains (Hajri et al., 2010) was used. Signals at the expected sizes were obtained suggesting a complete flagellar cluster in all strains, excepted in CFBP878, which gave no signal with *fleQ, fliE*, and *fliM* primers (Table 2). We also compared the N-terminal FliC sequences with the flagellin conserved domain Flg22, which is known as a major pathogen-associated molecular pattern (PAMP), activating host defense responses (Felix et al., 1999; Navarro et al., 2004; Shi et al., 2015). Nonpathogenic strains possessed the conserved Flg22 epitope whereas CFBP 2528 and CFBP 7179 had a different peptide, with a polymorphism in 7 amino acids (Figure 8). One of these six residues—aspartic acid (D)—has been shown in *X. campestris pv campestris* to be critical for elicitation activity in Arabidopsis (Sun et al., 2006). Its replacement by valine (V) in *X. campestris pv campestris* eliciting strain suppress the elicitation activity. In our *X. arboricola* pv *juglandis* (pathogenic) strains, the D residue is replaced by a V one (Figure 8).

### The four strains share a different repertoire of genes encoding a type IV secretion system (T4SS) and type IV effectors (T4Es)

**Figure 9:**
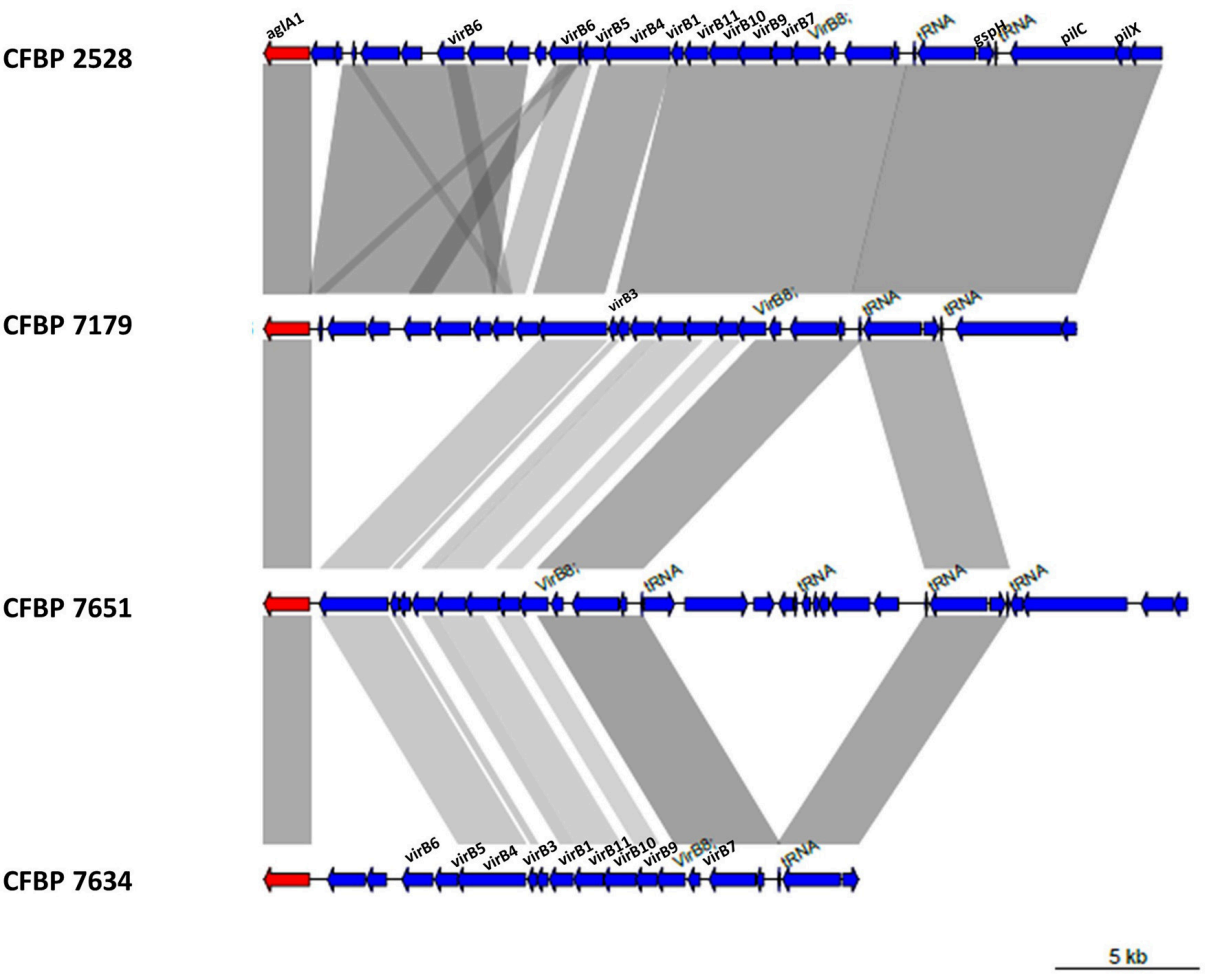
**Schematic representation of T4SS genes of the four ***X. arboricola*** strains**.

In *X. arboricola*, the T4SS encoding genes are approximately organized as in *X. citri* subsp. *pv citri* (Jacob et al., 2014) considering the fact that some proteins are not conserved (Figure 9). *VirB3* gene is absent in CFBP 2528. The protein encoded by this gene is thought to be involved in the production of the inner-membrane pore. VirB5 is lacking in CFBP 7651 in consequence of genomic rearrangements in this region and deletion of several genes including *virB5*. This gene encodes a pilus-tip adhesin. Additional genes coding proteins predicted to be involved in conjugative transfer were identified in the nonpathogenic strain, CFBP 7634. For instance, a set of CDS encoding proteins showed more than 92% identity by BLASTP with TrbB, TrbC, TrbD, TrbE, TrbJ, TrbL, TrbF, TrbG, TrbI from other *Xanthomonas* such as *X. gardneri* for the best score. These CDS are embedded in an MGE starting with an integrase/recombinase (XARJCFBP7634_b09150) and containing phagic genes, outer membrane efflux protein encoding genes, transcription regulator and pirin genes. A similar MGE also containing *Trb* genes was identified in the other nonpathogenic strain, CFBP 7651. This MGE also starts with a recombinase (XARJCFBP7651_a21800) but these two arrays of T4SS and MGE encoding genes are localized in different regions in the chromosomes.

So far, no T4E were described in *Xanthomonas*. Identification of T4Es based on T4Es already described in other species is difficult because of the expected low sequence similarity. The number of newly discovered effectors is increasing, but only in a limited number of species (e.g., *Legionella* or *Helicobacter*). According to Wang et al. (2014), amino-acid composition and amino-acid specific positions in C-termini of T4E sequences can be used to predict T4Es. The two models “T4SEpre_bpbAac” and “T4SEpre_psAac” of the T4SEpre package (Wang et al., 2014) were used here to predict T4Es in the four *X. arboricola* genomes. Only locus tags predicted by both models were retained (Table S6), as advised by Wang et al. (2014) to limit the false positive results. We observed that in a same orthologous group of T4Es, some of them were predicted by both models whereas others were predicted by the T4SEpre_psAac model alone (in bold and italic in the Table S6). The number of predicted T4Es was higher in pathogenic strains (17 in CFBP 2528 and 18 in CFBP 7179) than in CFBP 7634 (14 predicted T4Es) and in CFBP 7651 (10 predicted T4Es). Predicted T4Es specific to the pathogenic strains were localized in region corresponding to mobile genetic elements (near transposases) or in region with low GC% that were probably acquired by LGT. Among them, besides hypothetical proteins, one putative T4E is a cytochrome c-type subunit, as predicted by Wang et al. (2014) in *Salmonella*, and another one is a transcription repressor DNA-binding protein.

### Pathogenic strain genomes encodes specifically two non-fimbrial adhesins, FhaB and YadA-like

Bacterial attachment to the host surface is mediated by adhesins that are non-fimbrial (autotransporters; filamentous haemagglutinin-like proteins) or fimbrial (including type IV pili) adhesins, and both can contribute to virulence (Soto and Hultgren, 1999; Darsonval et al., 2009; Das et al., 2009; Gottig et al., 2009). The repertoires of genes coding non-fimbrial adhesins varied between the pathogenic strains and the nonpathogenic strains.

Two adhesin encoding genes were identified only in pathogenic strains. *yadA*-like and *fhaB* (Table S7). A *yadA*-like CDS was specifically found in pathogenic strains (GROUPORTHO3996), with the predicted domains serralysin-like metalloprotease C-terminal, trimeric autotransporter adhesin, YadA-like C-terminal. The predicted protein was 772-aa-long protein. This *yadA* sequence was associated in the two pathogenic strains with three other CDS encoding a S8 peptidase, a histidine kinase and a signal transduction response regulator, from the *cheY*-like family. In the corresponding regions of the nonpathogenic strains, five CDS encoding proteins with unknown function and a transcriptional regulator, one *ton*B-dependent receptor precursor, and one nuclease were found. One homolog of *fhaB* was predicted (GROUPORTHO3859) to encode a 4308- and 4034-aa-long protein in pathogenic strains, CFBP 2528 and CFBP 7179, respectively. This protein had a N-terminal filamentous hemagglutinin domain and filamentous hemagglutinin repeats, and had also a N-terminal pectate lyase. This encoded protein showed 86% identity and 99% similarity with FhaB of *X. fuscans, Xanthomonas axonopodis* or *X. citri*. This CDS was near a predicted sequence of *fhaC* coding a hemolysin activation/secretion protein also specific to pathogenic strains. FhaC contained a polypeptide-transport-associated (POTRA) domain in N terminus. Two autotransporters, the monomeric, XadA, and the trimeric YapH, were encoded in the four genomes.

The four genomes also harbored several clusters that are predicted to be involved in the biogenesis of type IV pilus. Type IV pili (T4p) are surface filaments involved in different functions, such as twitching motility, adhesion, biofilm formation, natural transformation, pathogenicity, and immune escape (Mattick, 2002; Craig et al., 2004; Nudleman and Kaiser, 2004). The filament is composed of a major pilin PilA plus the minor pilins PilE, PilV, PilW, PilX, and FimU encountered in *P. aeruginosa* and in the type IVa system, which is the system encountered in *Xanthomonas* (Burrows, 2012; Dunger et al., 2014). Recently, the organization of *pil* genes have been described in *X. citri* subsp *citri* (Dunger et al., 2014). In CFBP 7179 genome sequence, there was no predicted protein for PilA in the cluster (Table S8). Minor pilin PilX was absent in CFBP 7651 and PilV was absent in the pathogenic strains.

No T6SS was identified after BLASTN with known T6SS genes from Xcv85-10, Xff4834R, Xoo PXO99A against the four *X. arboricola* genomes.

### One chemosensor is specific of pathogenic strains

For chemotaxis sensors, i.e., MCPs, slightly differential repertoires were observed between pathogenic and nonpathogenic strains (Table S9). Two MCPs (GROUPORTHO78, GROUPORTHO3512) were present in CFBP 2528, CFBP 7179, CFBP 7651 but absent in CFBP 7634 probably following genomic rearrangements leading to the deletion of the gene in CFBP 7634. Two other MCPs (GROUPORTHO4206, GROUPORTHO4237) were present in CFBP 7651 and CFBP 7634. Moreover, additional MCP were specific of each strain. One homologous CDS encoding a MCP was present in each of the two pathogenic strains (XARJCFBP2528_d01320; 733 aa and XARJCFBP7179_e01340, 733 aa) but was absent in the two nonpathogenic strains. In CFBP 7634 genes encoding for an integrase (XARJCFBP7634_b11370) and phagic proteins were found at the same location. In CFBP 7651, two CDS were predicted (XARJCFBP7651_a35490 and XARJCFBP7651_a35500), which each corresponded to the C-terminal and N-terminal fragments of this MCP identified in the pathogenic strains. This MCP was therefore nonfunctional in CFBP 7651.

**Figure 10:**
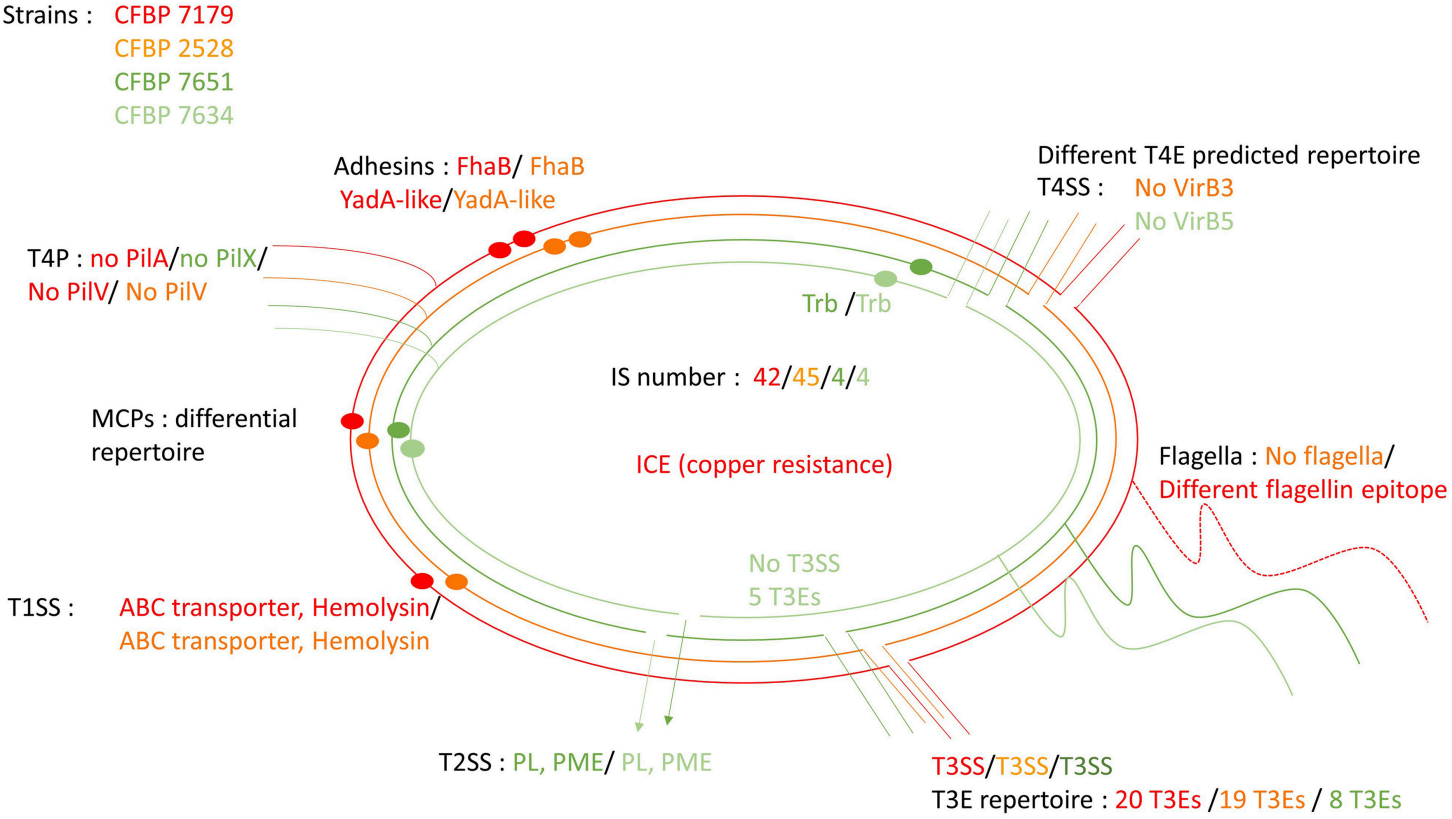
**Schematic representation highlighting conserved and differential features between the four genome sequences**. Specific features are written using the color code: red for CFBP 7179; orange for CFBP 2528; dark green for CFBP 7651, light green for CFBP 7634.

The Figure 10 highlights common and differential features of the four strains.

## Discussion

### Features of the genome sequencing

Each genome length was very similar to other strains belonging to this species (Caballero et al., 2013; Vandroemme et al., 2013a). It is noticeable that smallest *X*. *arboricola* genomes corresponded to nonpathogenic strains lacking the *hrp*-island (this study and Vandroemme et al., 2013a). The high GC content was a common characteristic of most genera within the *Xanthomonadacae* family (Saddler and Bradbury, 2005). ANI values were all above the threshold of the species level (Konstantinidis and Tiedje, 2005) and supports the grouping of the four strains within *X. arboricola*. This is in accordance with previous study (Essakhi et al., 2015).

### Mobile genetics elements

#### IS

The IS number observed in pathogenic strains was similar to the one found in *X*. *campestris* pv *raphani* (Bogdanove et al., 2011). But the number and the diversity of IS elements can be larger in other *Xanthomonas* such as *X. oryzae* (Salzberg et al., 2008) with up to 245 elements distributed in six families, or such as *Xanthomonas fragariae* with up to 420 elements representing at least seven families (Vandroemme et al., 2013b). The IS families are distinct accross *Xanthomonas* pathovars and species (Bogdanove et al., 2011). Most IS elements in our *X. arboricola* strains belonged to the IS3 and IS4 families (Table S3), which are also common families in *X. oryzae* and *X. campestris* genomes (Bogdanove et al., 2011). Similarly to *X. arboricola* genomes, in *X. campestris* pv. *vesicatoria* and *X. axonopodis* pv *citri*, the IS3 family is also highly abundant, whereas in *X. campestris* pv *campestris* and in *X. fuscans* subsp. *fuscans*, most IS elements belong to the IS5 family (Thieme et al., 2005; Darrasse et al., 2013). IS21 and Tn3 transposase which are unique to CFBP 7179 in *Xanthomonas* genus, are located on an ICE (see below). Previous genomic and genetic studies have established that ISs are a major and powerful force in genome evolution. The presence of multiple copies of an IS in a genome can trigger intragenomic homologous recombination, resulting in genome rearrangements (inversions) or deletions of the intervening genomic region (Salzberg et al., 2008), and interruption of genes, operons, or transcriptional signals (Schneider and Lenski, 2004; Darrasse et al., 2013). Organisms harboring ISs are thus subject to a variety of mechanisms that enhance genomic plasticity. In the two pathogenic strains ISs or transposases were found in the vicinity of several accessory genes like T3Es linked to host specificity. The two nonpathogenic strains have 10 times less ISs than their pathogenic counterpart. A similar observation was previously reported for *Yersinia pestis* compared to *Yersinia pseudotuberculosis*, its ancestral species (Parkhill et al., 2001) and IS expansion was found to be linked to niche specialization in several bacteria (Mira et al., 2006). Genome sequencing has revealed that some genomes contained large numbers of ISs, while others had none at all but Touchon and Rocha (2007) found no association between IS frequency and pathogenicity. ISs can be transferred between genomes by LGT mechanisms (Frost et al., 2005). Iranzo et al. (2014) suggested that the LGT rate might be determined by the bacterial ecological niches. But the abundance of IS copies could be driven by duplication-deletion mechanism (Iranzo et al., 2014). It would be interesting to compare a higher number of genomes in the *X. arboricola* species to confirm the IS number differences between pathogenic and nonpathogenic strains and to try to reveal if mechanisms are used by IS to choose a target (Siguier et al., 2014).

#### ICE and copper resistance

We showed that strain CFBP 7179 and 36 *X. arboricola* pv. *juglandis* strains harbor an ICE with copper resistant genes and are actually copper resistant. Seven strains showed copper resistance without positive *cop* gene detection by PCR. We can't rule out that these strains present sequence variations at primer sites preventing their amplification but a mechanism for copper resistance independent of ICE located *cop* genes could exist in these strains. Indeed, Gardan et al. (1993) have previously shown that copper resistance in strain CFBP 1022 (one of the seven Xaj-ICE negative strains) was linked to the presence of a plasmid of 111 kb. Consequently, we can hypothesize that copper resistance in strains lacking the ICE is associated to a plasmid absent in genomes that were sequenced. In *X. campestris* pv *campestris*, only plasmid-borne *cop* genes are essential for copper resistance. Nonetheless, homologs of these plasmid-borne copper resistance genes are present in the chromosomes of copper-sensitive and -resistant *Xanthomonas* (Behlau et al., 2011). In *X. axonopodis* pv *vesicatoria*, expression of *copAB* cluster (putative copper binding proteins) is regulated by CopL, and the corresponding gene is located immediately upstream of *copAB* (Voloudakis et al., 2005). No homolog of *cop*L was found in our four genomes which suggests that in *X. arboricola, copAB* regulation may be different. Copper is widely used in agriculture but the efficacy of copper is now reduced by the occurrence of copper-resistant strains in *Xanthomonas* (Behlau et al., 2011; Araújo et al., 2012) or *Pseudomonas* (Nakajima et al., 2002) species. The presence of this ICE in CFBP 7179 represents an example of probably environmental driven expansion of a bacterial genome because of a high selective pressure due to the extensive use of copper. Indeed Xaj-ICE has been only retrieved in strains responsible for recent epidemics in France (Hajri et al., 2010). The acquisition of this element probably conferred a selective advantage to these strains. Most CDS of the Xaj-ICE showed high identity with genes from *P. aeruginosa* strains and *S. maltophilia* strain D457, which belongs to the *Xanthomonadaceae* familly. It suggests that the Xaj-ICE should have been taken up by lateral transmission into the CFBP 7179 genome from a different genus donor strain. Transfers between distantly related genomes exist even if genome sequences dissimilarity is a barrier to LGT (Popa et al., 2011). It is interesting to notice that Xaj-ICE is the first ICE detected in *Xanthomonas* to date.

#### Other MGEs

The higher number in prophage in pathogenic strains could suggest a higher sensitivity to it but it should be assessed with other genomes. According to the evolutionary scheme proposed by Gillings et al. (2005) for the integron, we can hypothesize that genetic rearrangements in the cassettes of this integron could have accompanied niche specialization of our strains after the loss of activity of the integrase. How do the cassette arrays determine the ecological niche of each strain? This is currently unknown because most genes carried by the integron code for proteins with unknown activities.

### Secretion systems

#### T1SS

Hemolysins are of great importance for the pathogenesis in the host organism (Kanonenberg et al., 2013). CDSs linked to hemolysin secretion or hemolysin were only present in pathogenic strains. We hypothesized that these CDS were acquired by the common ancestor of the *X. arboricola* pv. *juglandis* strains by recombination. To our knowledge, no *Xanthomonas* hemolysin mutant is described to date: it would be interesting to realize functional analyses to study the role of these CDSs in pathogenicity.

#### T2SS

The absence of a *cbhA* ortholog in the four *X. arboricola* genome sequences is in agreement with their known inability to colonize xylem vessels. The gene *cbhA* is conserved in the xylem-invading *Xanthomonas* species (*X. albilineans, X. oryzae* pv. *oryzae, X. campestris* pv. *campestris, X. campestris* pv. *vasculorum*, and *X. campestris* pv. *musacearum*), but is missing in the non-vascular *Xanthomonas* species (*X. oryzae* pv. *oryzicola, X. axonopodis* pv. *citri, X. axonopodis* pv. *vesicatoria*) (Pieretti et al., 2012). The *cbhA* gene was also shown to contribute to virulence of the xylem-invading pathogen *Ralstonia solanacearum* (Liu et al., 2005). Putative aminopeptidase and pectate lyase encoding CDSs were present in the nonpathogenic strains, while derived in pathogenic strains: we hypothesized that these fragments represented remnants of the genes present in the common ancestor of pathogenic and nonpathogenic strains. Similarly, as homologs of pectinesterase gene present in nonpathogenic strains, are found in genomes of pathogenic strains belonging to other *Xanthomonas* species, the most parsimonious hypothesis will be in favor of the loss of this gene in the common ancestor of the pathogenic strains CFBP 2528 and CFBP 7179. Putative pectate lyase and xylosidase homologs were only detected in pathogenic strains and seem to have been acquired in pathogenic strains by LGT. PCWDEs are carbohydrate-active enzymes that have been classified in different families based on homology criteria (http://www.cazy.org/, Cantarel et al., 2009). Pectin methylesterase (PME) catalyzes de-esterification of pectin to make substrates available for subsequent action by polygalacturonase and pectate lyase. These enzymes act in concert in pectin degradation. The ability to degrade pectin may facilitate pathogen invasion into the cells of host plants and is useful for pathogens in term of virulence (Hugouvieux-Cotte-Pattat et al., 2014). Although our *in vitro* tests may not detect all pectinase activities, pectinase activities were only observed for the nonpathogenic strains. It has obviously no role in disease process, but may participate in nutrient uptake by these bacteria *in planta*. Vorhölter et al. (2012) shown that oligogalacturonides generated by pectate lyase activity in a pathogenic interaction involving *X. campestris* pv *campestris*, could elicit plant defense reactions. The two pathogenic strains which have a putative pectate lyase but undetectable PL activity, could have evolved to avoid production of PAMPS by PL activity.

#### T3SS

The *hrp-hrc* region and the *hrpF* peninsula were absent in the nonpathogenic strain CFBP 7634 however five T3E genes were retrieved in its genome. Given that HrpF functions as a translocon of effector proteins into the host cell (Rossier et al., 2000; Büttner and Bonas, 2002), we can assume that CFBP 7634 T3Es could not be translocated into plant cells. Previous studies showed that mutation of the *hrpF* locus of *X. oryzae* pv. *oryzicola* strain resulted in the loss of pathogenicity in rice and the inability to induce HR in non-host tobacco (Zou et al., 2006). Similarly, mutations in *hrpF* of *X. c*. pv. *vesicatoria* strain or *X. axonopodis* pv. *glycines* strain resulted in strains that were nonpathogenic in host plants and unable to elicit race-specific HRs (Rossier et al., 2000; Kim et al., 2003).

#### T3Es

Pathogenic strains presented a repertoire of T3Es, which was moderately large in comparison to other xanthomonads (Hajri et al., 2009). The presence of *awr4, sfrJ* and *xopAR* highlighted the limits of the PCR compared to genome sequencing. *sfrJ* is secreted through SPI2 in *Salmonella* (Cordero-Alba et al., 2012). However, these authors suggested a SPI2 independent role in environment as *sfrJ* is also present in a commensal *E. coli* strain devoided of T3SS. Differential T3Es in pathogenic strains (*xopAH* in CFBP 2528; *xopAI* and *xopB* in CFBP 7179) were probably acquired by different mechanisms (homologous recombination or LGT). Transcription activator-like (TAL) effectors were not detected in the four genome sequences but HiSeq technology is not the method of choice to detect these TAL effectors, because of internal repeats. However, Hajri et al. (2012) only detected *avrBs3* by PCR in the pathovar *corylina*.

#### Flagella

CFBP 2528 is impaired in motility because of a loss of *fliS* and *rpoN* CDSs. Mutant affected in *fliS* still produce functional flagella in *Salmonella* (Yokoseki et al., 1995) whereas *rpoN* mutant has been shown to loose motility in *X. oryzae pv oryzae* (Tian et al., 2014). Darrasse et al. (2013) also reported a lack of motility in other *X. arboricola* strains, such as pathovar *corylina* type-strain CFBP1159. We suggest that the modifications observed for the flagellin epitope in both pathogenic strains affect flagellin perception *in planta* and could prevent recognition of pathogenic strains at an early stage of infection as already observed for other *Xanthomonas* (Sun et al., 2006). This could be a mechanism of evolution to avoid PAMP-triggered immunity as previously suggested for other *Xanthomonas* strains (Jacobs et al., 2015).

#### T4SS and T4Es

The T4SS translocates DNA and proteins to bacterial or eukaryotic target cells by a direct cell-to-cell contact (Christie et al., 2014). A *virB* cluster was found in the four *X. arboricola* strains. *VirB3* gene is absent in CFBP 2528. The protein encoded by this gene seems to be essential for pilus assembly and substrate translocation (Guglielmini et al., 2014). VirB5 is lacking in CFBP 7651. This gene encodes a pilus-tip adhesin that could initiate contact with host cells (Backert et al., 2008). An additional T4SS locus with *Trb* genes was found in nonpathogenic strains in different regions in the chromosomes, near a recombinase, suggesting independent LGT events in these two nonpathogenic strains. As no T4E were described so far in *Xanthomonas*, we used the method of Wang et al. (2014) to predict T4Es. Among 10 to 18 T4Es detected *in silico*, a transcription repressor DNA-binding protein was found in pathogenic strains: this latter predicted T4E is perhaps interesting as T4Es can manipulate host pathways for a survival strategy (Hubber and Roy, 2010). Nevertheless, these proteins need further experimental validation analysis. It should be noted that in *X. citri* subsp. *citri* T4SS is not induced under infection conditions (Jacob et al., 2014).

### Adhesion and chemotaxis

#### Adhesion

One homolog of *fhaB* was detected only in pathogenic strains. FhaB seems to be involved in the colonization of both the leaf surface and the apoplast in *X. citri* subsp. *citri* (Gottig et al., 2009). However, other non fimbrial adhesins (XadA and YapH) are encoded in the four genomes. The absence of CDSs encoding PilA in CFBP 7179, PilX in CFBP 7651 and PilV in both pathogenic strains suggests that T4p biogenesis is probably impaired in these strains (Nguyen et al., 2015). It could be interesting to conduct *in vivo* adhesion analysis in order to observe behaviors of the strains.

#### Chemotaxis

The repertoire of MCPs was different between strains with MCPs specific of pathogenic or of nonpathogenic strains. MCPs, which are cell membrane-bound chemoreceptors, are involved in the detection of molecules such as attractant or repellant. Subsequent movement of the cell through flagellar motility allow bacteria to go toward or away from perceived molecules (Vladimirov and Sourjik, 2009). Ability to specifically detect a molecule could allow the pathogenic strains to colonize environment that could remain inaccessible for nonpathogenic strains. This suggested different chemotaxis properties. Characterizing repertoires of MCPs in a large collection of strains and functional analyses would be interesting to further study the role of the different MCPs in the plant colonization.

## Conclusion

Differences between the two pathogenic strains, CFBP 2528 and CFBP 7179, and the two nonpathogenic strains, CFBP 7634 and CFBP 7651, all isolated from the same plant species, i.e., walnut, concerns a full range of functions involved in ability to colonize plants from sensing of the environment and to cross-talk with the immune system. Several non-fimbrial adhesins and one hemolysin may allow pathogenic strains to adhere or aggregate more efficiently than nonpathogenic strains to plant tissues or to form more stable or resistant biofilms. Differential repertoires of PCWDEs between pathogens and commensals could allow the colonization of separate niches. A larger repertoire of T3Es in pathogens may be an efficient means to interfere with plant immune system allowing ingress and multiplication inside plant tissues, but also can significantly contribute to growth. One chemoreceptor was specifically identified in pathogenic strains and might allow pathogens to differentially perceive the environment. We highlighted a larger set of various mobile genetic elements in pathogen genomes and different genome organizations, which were driven by recombination events or horizontal transfers. We propose that these events were closely related to bacteria encountered in their physical environment rather than to phylogenetically related bacteria. From these genome comparisons it is not possible to answer the question of the origin of these strains. Do pathogenic strains evolve from nonpathogenic ancestor through acquisition of pathogenesis-associated genes or in contrast do nonpathogenic strains evolve from pathogenic ones through the loss of energetically costly functions? Bacteria live in interaction with their biotic environment and evolve in dynamic microbial communities, which may act as reservoir of genes and also favor loss of genes by providing mobile genetic elements. A better understanding of emergence of pathotypes and finally diseases may arise from the deciphering of whole microbial communities.

### Conflict of interest statement

The authors declare that the research was conducted in the absence of any commercial or financial relationships that could be construed as a potential conflict of interest.
